# Supplementary material for: Probabilistic analysis of COVID-19 patients’ individual length of stay in Swiss intensive care units
Source: PLoS One. 2021 Feb 19;16(2):e0247265. doi: 10.1371/journal.pone.0247265 (PMC7894868; doi:10.1371/journal.pone.0247265)
Supplement: S1 Appendix — (PDF) [file pone.0247265.s001.pdf]

S1 Appendix:

Probabilistic analysis of COVID-19 patients' individual length of stay in Swiss intensive care units

Alexander Henzi[1], Gian-Reto Kleger[2], Matthias P. Hilty[3], Pedro D. Wendel Garcia[3], RISC-19-ICU Investigators for Switzerland[3], Johanna F. Ziegel[1]

1 Institute of Mathematical Statistics and Actuarial Science, University of Bern, Switzerland

2 Division of Intensive Care Medicine, Cantonal Hospital, St.Gallen, Switzerland

3 The RISC-19-ICU registry board, University of Zurich, Switzerland and Institute of Intensive Care Medicine, University Hospital of Zürich, Switzerland

January 6, 2021

S1 Fig. Illustration of the relation between admission date, LoS, update time and snapshot date. For patients discharged before the update time, the (uncensored) LoS is available. The censored LoS is available only if a patient is discharged after the snapshot date, but not if the patient left the ICU between the update time and the snapshot date.

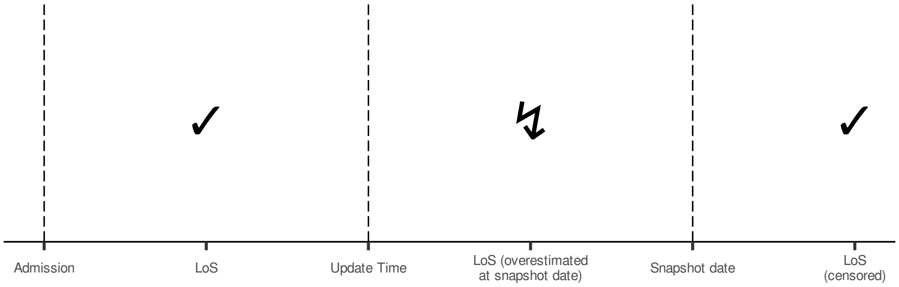

A. Censoring of LoS in the COVID-19 dataset

Since the COVID-19 pandemic is ongoing, it is likely that the set of patients in the RISC-19-ICU registry with available LoS has a selection bias towards shorter LoS. The natural approach to deal with this problem would be to treat the patients with missing LoS as censored observations with censoring time the number of days between admission and snapshot date. Unfortunately, this approach appears to be misleading and overestimates the LoS for the following reason. The data for each patient in the RISC-19-ICU registry is updated periodically by the corresponding ICU. We call the date of the last update for a given patient the update time. If the patient's LoS in the ICU has terminated before the update time, then we observe the LoS, if the patient is still in the ICU at the snapshot date, then the LoS is censored as above. However, there is the possibility that the patient has left the ICU between the update time and the shapshot data, and there is no possiblity to see this from the data. S1 Fig illustrates this problem, and S2 Fig shows how many patients are subject to this issue.

B. Evaluation of probabilistic predictions

Probabilistic predictions should be calibrated and sharp [1]. Calibration refers to the statistical compatibility of predictions and observations, and there are several tools available in the literature to assess calibration graphically and with statistical tests. The most prominent tool are so-called Probability Integral Transform (PIT) histograms, which are a histogram of $F_1(y_1), \ldots, F_n(y_n)$ [2,3]. Here, $(F_1, y_1), \ldots, (F_n, y_n)$ are a generic notation for the available prediction-observation pairs. Predictions are called probabilistically calibrated if the PIT histogram is flat, and there are strong arguments that probabilistic calibration is an essential requirement for probabilistic forecasts [4]. The notion of probabilistic

S2 Fig. Patient admission dates and LoS. Dots show the LoS of patients who already left the ICU before the snapshot date (June 15). Black crosses show the time between the admission and the snapshot date for patients for which no discharge time is available in the database.

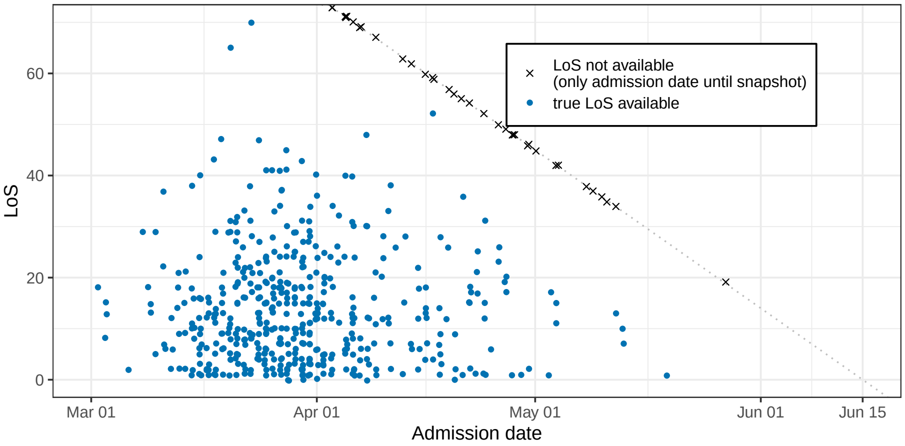

calibration has been reintroduced in under the name of D-calibrated in [5]. Probabilistic predictions are called marginally calibrated if $(1/n)\sum_{i=1}^{n}\mathbf{1}\{y_i \le y\} = (1/n)\sum_{i=1}^{n} F_i(y)$ for all $y \in \mathbb{R}$, that is, the observed frequency of realizations of Y below any threshold y should be equal to the average prediction of this frequency [4].

Calibrated probabilistic predictions are not necessarily informative. Therefore, the authors of [1] postulated the principle that probabilistic predictions should *maximize sharpness subject to calibration.* Sharpness is a property of the forecasts only and it refers to how concentrated the predictive distribution is. A forecast is sharper if it yields shorter prediction intervals. Proper scoring rules allow to assess sharpness and calibration of a forecast simultaneously [6]. A widely used example is the Continuous Ranked Probability Score (CRPS) which is defined as

$$\mathrm{CRPS}(F, y) = \int_{-\infty}^{\infty} (F(t) - \mathbf{1}\{y \le t\})^2 \mathrm{d}\, t.$$

for a CDF F and a real number y [7]. A forecast procedure is better the lower the average realized CRPS

$$\frac{1}{n}\sum_{k=1}^{n} \mathrm{CRPS}(F_k, y_k).$$

The significance of differences in forecast performance can be assessed by a Diebold-Mariano test [8].

S3 Fig. PIT histograms for the ECDF and the DIM predictions.

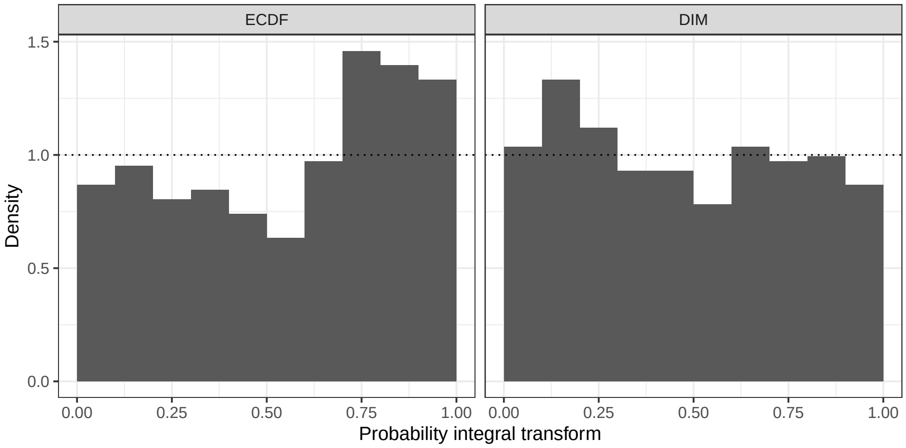

C. Diagnostic plots for calibration of DIM predictions

S3 Fig shows the PIT histograms for the ECDF predictions and the DIM predictions.

D. Figures on LoS by regions

S4,S5,S6 Figs summarise the COVID-19 dataset and the corresponding predictions split up by regions in Switzerland.

References

1. Gneiting T, Balabdaoui F, Raftery AE. Probabilistic forecasts, calibration and sharpness. J R Stat Soc Series B Stat Methodol. 2007;69:243–268.
2. Dawid AP. Statistical theory: The prequential approach. Journal of the Royal Statistical Society: Series A. 1984;147:278–290.
3. Diebold FX, Gunther TA, Tay AS. Evaluating density forecasts with applications to financial risk management. International Economic Review. 1998;39:863–883.
4. Gneiting T, Ranjan R. Combining predictive distributions. Electronic Journal of Statistics. 2013;7:1747–1782.
5. Andres A, Montano-Loza A, Greiner R, Uhlich M, Jin P, Hoehn B, et al. A novel learning algorithm to predict individual survival after liver transplantation for primary sclerosing cholangitis. PLOS one. 2018;13(3):e0193523.

S4 Fig. (a) Empirical distribution of LoS of COVID-19 patients in the regions NE and WT. (b) QQ-plot of the empirical distributions.

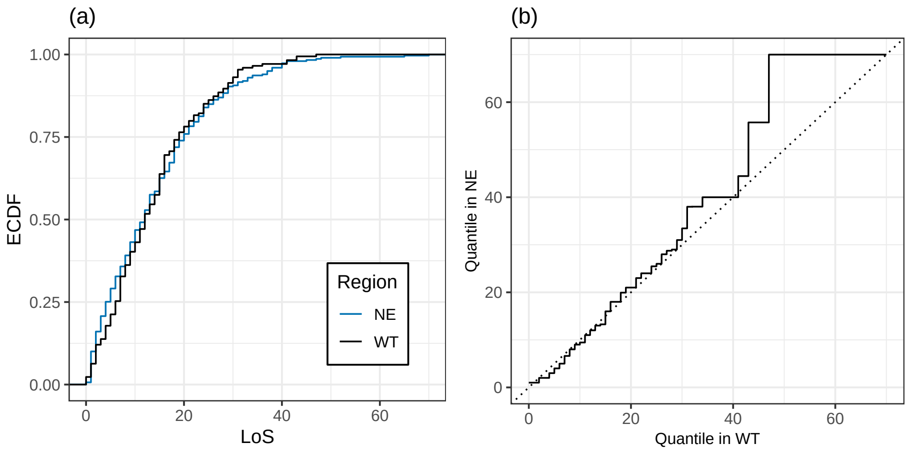

S5 Fig. Empirical LoS distributions of COVID-19 patients and corresponding DIM forecasts for the regions NE and WT. The DIM forecasts are as in Fig 2 in the article.

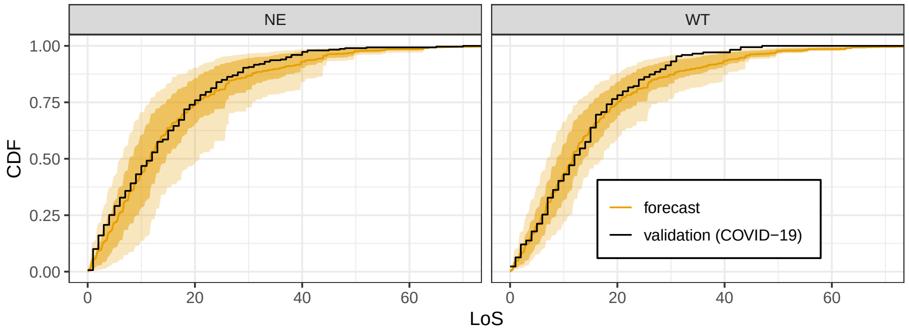

S6 Fig. DIM forecasts for COVID-19 patients, depending on region.

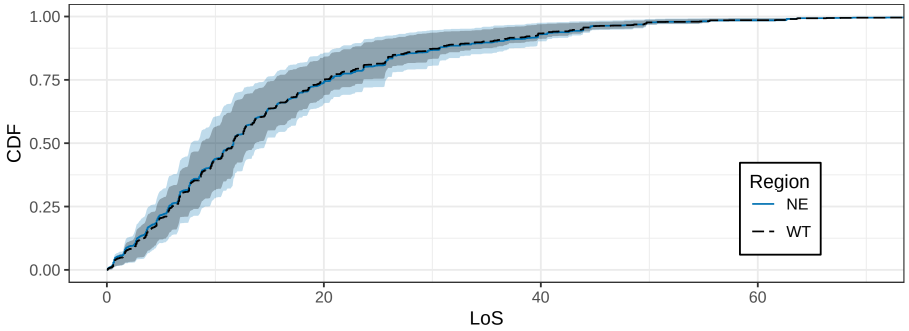

6. Gneiting T, Raftery AE. Strictly proper scoring rules, prediction, and estimation. Journal of the American Statistical Association. 2007;102:359–378.

7. Matheson JE, Winkler RL. Scoring rules for continuous probability distributions. Management Science. 1976;22:1087–1096.

8. Diebold FX, Mariano RS. Comparing predictive accuracy. Journal of Business & Economic Statistics. 1995;13:253–263.
