# Supplementary material for: Probabilistic analysis of COVID-19 patients’ individual length of stay in Swiss intensive care units
Source: PLoS One. 2021 Feb 19;16(2):e0247265. doi: 10.1371/journal.pone.0247265 (PMC7894868; doi:10.1371/journal.pone.0247265)
Supplement: Attachment — Submitted filename: response_Covid19_2021.01.06.pdf [file pone.0247265.s004.pdf]

Response to the reviewers' comments on
"Probabilistic analysis of COVID-19 patients' individual length of stay in Swiss intensive care unnits"

by Alexander Henzi, Gian-Reto Kleger, Matthias P. Hilty and Pedro D. Wendel Garcia on behalf of RISC-19-ICU Investigators for Switzerland, Johanna F. Ziegel

We are grateful to the review team for the constructive reports and helpful comments on our paper. To distinguish between the decision letter/review reports and our responses, the *editor's and reviewers' comments* are in *italics*, whereas our responses are in normal font.

Journal requirements

Please ensure that your manuscript meets PLOS ONE's style requirements, including those for file naming. The PLOS ONE style templates can be found at https://journals.plos.org/plosone/s/file?id=wjVg/PLOSOne_formatting_sample_main_body.pdf and https://journals.plos.org/plosone/s/file?id=ba62/PLOSOne_formatting_sample_title_authors_affiliations.pdf

We have consulted the style requirements and adapted our manuscript accordingly.

In your ethics statement in the manuscript and in the online submission form, please provide additional information about the patient records/samples used in your retrospective study. Specifically, please ensure that you have discussed whether all data/samples were fully anonymized before you accessed them. If patients provided informed written consent to have data/samples from their medical records used in research, please include this information.

We have clarified this point in the manuscript by adapting lines 87–88 to
"Fully anonymized datasets, in regard to Swiss law, were collected using a secure REDCap infrastructure provided by the Swiss Society of Intensive Care Medicine."
The online submission form as been updated accordingly.

In your Data Availability statement, you have not specified where the minimal data set underlying the results described in your manuscript can be found. ...
We have updated our Data Availability statement and are now compliant with PLOS journal requirements. In particular, in the supplement S2 Data, we provide a minimal dataset which allows to reproduce the figures, tables, and results in the article. Additionally, we provide code that demonstrates the computation of the statistical models and the usage of LoS predictions (supplement S3 Code).

Response to the Editor

The paper is based on an interesting topic, the statistical analysis has been performed appropriately, but there many concerns authors should correct and some concepts clarified to make the manuscript suitable for publication. This paper could be very difficult to be interpreted by a physician without mathematical experience. Authors should simplify the interpretations of results.

Thank you for your positive overall assessment of our work. In the revised version of the manuscript, we have addressed all issues raised by the reviewers. In particular, we have given more intuitive and concrete explanations of the goals and conclusions of our results.

Response to Reviewer 1

This paper attempts to provide a way to estimate LoS for individual patients, based on characteristics available within the first 24 hours of admission. I think that this is a very valuable contribution, as it can help with health care planning and provide more accurate estimates for when hospital capacity may be exceeded.

Thank you for your positive assessment of our work.

Although I think this is a nice paper in principal, I don't think enough information has been provided for me to adequately review the methods and results. As it stands I don't think the methods are clear, and the interpretation of the results is difficult to follow. I have tried to highlight below areas that I think could be made clearer:
Line 13, you talk about probabilistic predictions, but even with your definition it is not clear what this means. How is the uncertainty of the LoS quantified? This ties in with Figure 1, which I also don't think is clear. There is very little information provided in the figure legend or text about figure 1, and it is difficult to interpret. As I understand, each vertical line indicates how long someone actually stays in hospital, so patient 4 stayed in for 40 days? And then patient 3 was released from ICU on day 1, but had a very low possibility of doing so (0.05)? I think more of an explanation is the figure legend and text is required to adequately explain this figure, maybe even just proving a small example as I have done above would help. I think it's a nice figure which if adequately explained in the legend and text would help to clarify the aim/ methods of the paper. At the moment I don't think your overall aim is clear.

Concerning line 13: The predictive CDF quantifies the uncertainty of the LoS comprehensively since it is an estimate of the conditional distribution of the LoS. Mathematically, the conditional distribution of the LoS *is* its uncertainty. We understand that this definition does not give enough intuition. Therefore, we have added that, in particular, the predictive CDF allows to give prediction intervals with any desired coverage probability.
Concerning Figure 1: We have extended the explanation of Figure 1 and made it more intuitive, both in the text and in the figure caption.

It is also not made clear in the introduction how having the CDF would be used. Who are you expecting to use your results, and how? It says in the abstract that individuals with long LoS could be discovered early, indicating that maybe this is for hospital planning purposes? Also, going back to figure 1, patient 4 has the longest LoS, but it is not clear precisely what indicates that patient 4 is going to have the longest LoS from the Forecast CDF. So difficult to see how the CDF is going to indicate long los.

To address this point, we have added specific illustrative examples for the use of the predictive CDF starting in the second paragraph of page 3. In particular, we clarify how the CDF can be used to derive alerts for patients that are likely to have a long LoS. The examples can also be tested with new code that we provide; see the next comment for details.

Methods:

I understand that you are not able to release your data, however, there is no reason that the code could not be released, along with a brief description of the datasets you have available

(could even consider creating some dummy datasets). This would allow others to understand your methods more clearly, and be able to repeat your analysis.

In a Supporting Information file (S3 Code), we have now made available code and a simulated dataset with the same variables as used in the paper. We hope that this allows to more easily understand the analysis and methods. In the code, we also illustrate how to derive information from predictive CDFs.

From your methods, it's not adequately explained what the difference is between ECDF and CDF. In addition, in the results you discuss ECDF, and show plots comparing them to CDF, but I think providing a brief summary at the beginning of the results would aid interpretation.

We added a further sentence explaining the ECDF forecast in Section 3.1.

Results:

You talk about how this is done on an individual level, and yet in Figures 2-4 your provide figures for one overall CDF. So is this a CDF for the whole dataset? How have you combined them?

In the description of Fig 2. it is explained that the CDFs are averaged pointwise. This means, the curves show the vertical average of the predictive CDFs for all single patients (like the four curves in Figure 1) in the COVID-19 dataset. To indicate the variability of the individual patients' CDFs, shaded bands show the pointwise quantiles of the CDFs. In the new sample code in supplement S3 Code, it is demonstrated how the aggregated CDFs and the bands are computed. We have now expanded the figure caption to give a better explanation.

Line 138 you talk about panel c of figure 2, but there is no panel c.

Thank you for spotting this typo. It should be panel (b) and is now corrected.

Overall, I think this is an interesting idea and concept, but in it's current format I don't think their methods are reproducible and their results are not easy to follow. I think they need to be clear who is there target audience, is it for mathematical modellers or people with a more clinical background? Clinicians or anyone with a non-technical background would struggle to know what to do with this information. However, I do think it is highly relevant, so I hope that the authors are able to revise their manuscript to make things clearer. I think being able to predict who is going to spend a long time on ICU is of great value. Best of luck with the submission.

Thank you. We have done our best to make the material more accessible. In a modern hospital setting and especially in a health crisis like the current COVID-19 pandemic, a multiprofessional framework is of crucial importance. The frontline clinicians in the ICU who have to triage patients, as well as ICU directors should collaborate with epidemiologists, infectious disease specialists, disease modellers, mathematiciens and data scientists. Multiprofessional collaboration would allow to make more and faster progress in the future, especially in ICUs. Our goal is to demonstrate the benefits of probabilistic forecasts to potentially any reader who might use predictions for COVID-19 patients' LoS (mathematical modellers, clinicians, or decision makers) and has not thought about this problem from the perspective of probabilistic forecasting so far.

Response to Reviewer 2

In this paper the authors developed a new semi parametric distributional index model that should provide a probabilistic prediction of ICU length of stay 24 hours after admission for COVID-19

patients. The model is based on 4 covariates: age, gender, SAPS II and NEMS. According to the authors these parameters were the only possible choice. I wonder if and how covariates dependance affects the model. Particularly:

- *age is included in SAPS II*
- *both SAPS II and NEMS are expression of the severity of patients status, is this model performing better than a simpler model including just SAPS II?*

On the opposite, I wonder if including more specific variables, such as the coagulation status would provide better predictions.

There are indeed dependencies between the covariates, such as the ones you mention above. We argue that it is still useful to include all of them in the model. The variable age is contained in SAPS II as a discretized effect with 6 levels. But given the fact that age enters the model as a cubic regression spline with sufficiently high dimension, manually removing the age variable from SAPS II would essentially correspond to a basis transformation of the model and not affect the results. Clearly, it may affect the respective effect curves of age and SAPS II, but the exact attribution of the LoS to certain variables is of secondary importance, as we are mainly interested in precise predictions. The information provided by the NEMS is not redundant to SAPS II. NEMS is a crucial variable for COVID-19 patients since it contains information on the ventilation status, therapy with cardovascular drugs and renal replacement treatment, which are not in the SAPS II. More precisely than the SAPS II, the NEMS reflects the actual therapeutic intensity a patients needs, and it is therefore likely to be one of the earliest markers for LoS.

Concerning your remark on more specific variables, there are certainly many relevant predictors for COVID-19 patients. However, most of them concern mortality and not LoS. This includes coagulation status. These values are available in the RISC-19-ICU registry but not in the training data. We believe that a successful model for probabilistic predictions of LoS should rely values that are routinely recorded and available early after hospitalization. Clearly, the DIM model could be adapted at a later stage to variables specific to COVID-19 patients once that enough training data of COVID-19 patients is available.

For coagulation status in particular, it is not clearly understood to which extent classic coagulation parameters such as Thrombocytes, Fibrinogen or INR phenotype correlate with disease severity or LoS in COVID-19, as coagulation disorder in COVID-19 is likely to be associated with von Willebrands Factor and ADAMTS13. Regarding the frequently used D-Dimer value at the moment in COVID-19, its temporal variability is high and with the rise of therapeutic anticoagulation its overall predictive capacity remains to be conclusively assessed.

Thank you for drawing our attention to argue this point carefully. We have added these important arguments in the paper; see lines 143–153, 286–294.

For the quality and soundness of the statistical analysis, I do not have the skills to judge, and an experts opinion is needed. Minor comments: Minor language revision is needed, as the manuscript contains several typos

We have checked the manuscript for typos.

Response to Reviewer 3

The authors proposed a new semi-parametric model to predict individual ICU LOS even though prediction of LOS at the patient level is difficult and none of the available models were reli-

able. The model was fully demonstrated in two of method papers showing that it provides more information than classical models.

In Figure 1, predicted CDFs of LOS and the corresponding true LOS were depicted. In reality, patient 3 had the shortest LOS and patients 1 and 2 had longer LOS. However, the forecasted CDF for those three patients were very close to each other. It looks like the proposed model doesnt have the ability to provide satisfactory forecasts at the patient level.

It was difficult for us to address this point since it is vague what is meant by the 'the CDFs are very close to each other' and what are 'satisfactory forecasts at the patient level'. The forecasts we provide have been validated with respect to decision theoretically principled criteria for forecast evaluation. Predictive CDFs assess uncertainty comprehensively which means that it can happen that for some patients the predictive CDFs are more informative for others: There are cases when the LoS is very variable even given the covariates, whereas in other cases it can be predicted rather precisely. The strength of our proposal is that this information is inherent in the forecast.
The examples of four patients in Figure 1 were drawn at random.

The authors mentioned LOS of a patient in ICU also depends on the characteristics and policies of the ICU. The COVID-19 pandemic placed a significant burden on healthcare systems. It induced unprecedented strain on ICU resources. It brought systematic error of prediction by using patients who were diagnosed of ARDS in 6 concurrent years as the training cohort. Because both measured and unmeasured confounders wound be unbalanced between training and COVID-19 cohorts. It may be helpful to present comparisons of other measured confounders and patients dispositions between cohorts.

During the first wave of the pandemic in Switzerland in the spring of 2020 (which corresponds to the data used in this article), the health care system was not decompensated, and thus, the confounder of limited ICU resources is probably not of importance for this analysis.
There have been discussions how and if classical ARDS and ARDS secondary to COVID-19 (C-ARDS) are different. Initially, substantial differences were postulated (https://pubmed.ncbi.nlm.nih.gov/33197387/, https://pubmed.ncbi.nlm.nih.gov/32697492/, https://www.ncbi.nlm.nih.gov/pmc/articles/PMC7577365/pdf/134_2020_Article_6281.pdf) but more recently consensus is growing that C-ARDS is most probably similar to classical ARDS in treatment intensity and therapeutic approach (https://www.ncbi.nlm.nih.gov/pmc/articles/PMC7549087/pdf/134_2020_Article_6275.pdf). In view of this, as such the historical training data is as well chosen as historical data can be. Furthermore, the NEMS evaluates how severe or nursing intensive a patient is, independently of the diagnosis. Therefore, using it as a covariate in prediction is likely to mitigate confounders between training data and COVID-19 dataset.
Thank you for promting us to address these important issues. We have added these arguments in lines 273–284.

Patients who were admitted to ICU had two dispositions, discharged or dead. It looks like the authors included both discharged and dead patients in analysis. Was patients disposition considered differently in model? If not, can the authors clarify?
We did not treat discharged or dead differently in our model, since patient disposition is not important for logistic ICU considerations; a dead patient is, as out of the ICU as a surviving one. We are not aiming to provide a COVID-19 severity score. The model uses covariates that are available 24 hours after admission at the latest and models the time from admission to discharge, independently of the reason for the discharge.

In line 153, the authors reported patients who were greater than 80 years of age have much shorter LOS. Is it because in-ICU mortality rate was higher for this age-subgroup?

This statement refers to a comparison of LoS of COVID-19 patients of different age groups. Yes, indeed, patients above 80 years are normally much frailer and as such their response to insults is much more critical. It is highly likely that this represents an overall very high mortality of this sub-population, which has already been described in the literature (https://pubmed.ncbi.nlm.nih.gov/31517831/).

The authors used cubic spline for continuous variables age, SAPS II, and NEMS in regression. Can the authors justify their decision to use cubic spline and how the assumptions of such a model were considered? Did the authors conducted tests for curvature and tests for significance of each curve?

Cubic regression splines have been applied previously in the literature to model the effects of continuous variables in LoS predictions, for example in reference [4] cited in the paper. Our implementation uses the `mgcv` package in `R`, where the splines are *penalized* cubic splines which are automatically shrunk towards lower dimensional splines in case the dimension (order of polynomials, number of knots) is too high. This makes them suitable for fitting arbitrary smooth functions while at the same time preventing overfitting. Thanks to the penalization and due to the fact that we are mainly interested in prediction and not in estimation, we argue that it is not necessary to conduct tests for curvature or significance of the spline curves. We have added to the article that we are using *penalized* cubic splines.

Figure 1: The color and pattern of lines confused me. Why did you use green lines for patients 1, 2, and 3 and red lines for patient 4?
We have adapted the color scheme to avoid confusion. There was no intention to mark differences between patients by the colors since the four patients were drawn at random from the validation data.

The paragraph of censoring of LOS in Appendix should be reported as a limitation in the discussion section.
We have reported the censoring problem as a limitation in the discussion. It can be found in lines 294–303. The Online Data Supplement merely gives further details.
