# Supplementary material for: Probabilistic analysis of COVID-19 patients’ individual length of stay in Swiss intensive care units
Source: PLoS One. 2021 Feb 19;16(2):e0247265. doi: 10.1371/journal.pone.0247265 (PMC7894868; doi:10.1371/journal.pone.0247265)
Supplement: Attachment — Submitted filename: response_Covid19_2021.02.01.pdf [file pone.0247265.s005.pdf]

Response to the reviewer's comment on the revised manuscript
"Probabilistic analysis of COVID-19 patients' individual length of stay in Swiss intensive care units"

by Alexander Henzi, Gian-Reto Kleger, Matthias P. Hilty and Pedro D. Wendel Garcia on behalf of RISC-19-ICU Investigators for Switzerland, Johanna F. Ziegel

We would like to thank the review team for carefully reassessing the revised version of our paper. To distinguish between the decision letter/review reports and our responses, the *editor's and reviewer's comments* are in *italics*, whereas our responses are in normal font.

Response to the Editor

Most of comments have been addressed. I've asked a check to the statistician, see conclusions below. Please modify the manuscript as suggested to make it definitely suitable for publication.

Thank you for your careful consideration of our work. We have addressed the remaining comment of reviewer 3 as detailed below.

Response to Reviewer 3

The authors have addressed most of my comments and concerns.

We are glad that we could address most of your concerns satisfactorily with our revision.

The reviewer still worry about prediction accuracy. In figure 1, patient 3 had the shortest LoS but the forecast CDF curve was in the middle. The order of realized LoS from the shortest to the longest were (patient 3 < patient 2 < patient 1 < patient 4). But the predicted probabilities were in different order.

Yes, that is correct indeed. Please note that we claim and justify that the conditional LoS distributions are *stochastically ordered*, with respect to the index function. For example, the CDF F_1 of patient 1 is above the CDF F_2 of patient 2. This means that for all thresholds t the probability $1 - F_1(t)$ that patient 1 stays longer than t in the ICU is smaller than the respective probability $1 - F_2(t)$ for patient 2. This does *not* imply that every realization $X_1 \sim F_1$ from the CDF of patient 1 will be smaller than every realization $X_2 \sim F_2$ from the CDF of patient 2. Therefore, it is no contradiction or indication of a lack of prediction accuarcy if there are some patients where realizations are ordered differently than the predictive CDFs.
Figure 1 is just an illustration of how the predictive CDFs derived with the DIM model can look like. From these four randomly drawn examples, no sensible conlusion about prediction accuracy is possible.

In the legend of figure 1, you said if a patient left on day t, the predictive CDF would jump from 0 to 1 at t. However, the CDFs in figure 1 didn't do so. Patient left on day 1, but the corresponding CDF didn't jump to 1.

We admit that this sentence was possibly confusing. We have reformulated the Figure caption to make things more clear. For sake of completeness, let us explain the original sentence that has confused the reviewer. It read: "If we were certain that a patient leaves on day t, the

predictive CDF would jump from 0 to 1 at t." The predictive CDFs are generated *without* the knowledge of the realized LoS of the patient, that is, in the case of patient 1, we do not know at the time of prediction (when we created the corresponding CDF) that he/she will leave on day 20. If some oracle had told us at the time of prediction that patient 1 will leave on day 20, then we should have given a predictive CDF that jumps from 0 to 1 at $t = 20$. In absence of an oracle, the predictive CDFs encodes the inherent uncertainty of an unknown future outcome.

The reviewer suggested to draw a 2-D scatter plot to indicate prediction accuracy. The x-axis is the observed values of the LoS and the y-axis is the probability that the respective patient would discharged on the realized day. Each dot represents each patient. For example, patient 1 was discharged/dead on day 20 and the probability that he/she would discharged on day 20 was about 80%. So the dot of patient 1 should be located at ($x = 20$*,* $y = 80\%$*).*

Following the large statistical literature on the correct evaluation of probabilistic predictions, we have assessed prediction accuracy with proper scoring rules (specifically the CRPS) and with PIT histograms. Details and established references for these procedures are given in the Supporting Information S1 Appendix B. The plot suggested by the reviewer is unfortunately not a possible alternative. The conditional LoS distributions are continuous distributions, that is, the probability that the LoS of patient 1 takes a specific value is zero for any value, contrary to the statement of the reviewer. (For patient 1, the probability of staying *at most* 20 days in the ICU is 80%.) This is not a flaw of the model. (The same is true for any continuous distribution such as the normal distribution, the exponential distribution, gamma disributions, etc.)
However, PIT histograms are actually fairly similar to what the reviewer was probably hinting at. If the true conditional conditional distribution of the LoS X_1 of patient 1 is the predicitve CDF F_1, then $F_1(L_1)$ will have a uniform distribution on $[0, 1]$. It is exactly this uniformity that is checked with a PIT histogram.
